# Chronic Cerebrospinal Vascular Insufficiency Is Not Associated with HLA DRB1*1501 Status in Multiple Sclerosis Patients

**DOI:** 10.1371/journal.pone.0016802

**Published:** 2011-02-14

**Authors:** Bianca Weinstock-Guttman, Robert Zivadinov, Gary Cutter, Miriam Tamaño-Blanco, Karen Marr, Darlene Badgett, Ellen Carl, Makki Elfadil, Cheryl Kennedy, Ralph H. B. Benedict, Murali Ramanathan

**Affiliations:** 1 Department of Neurology, State University of New York, Buffalo, New York, United States of America; 2 Department of Neurology, Buffalo Neuroimaging Analysis Center, State University of New York, Buffalo, New York, United States of America; 3 Department of Biostatistics, University of Alabama, Birmingham, Alabama, United States of America; 4 Department of Pharmaceutical Sciences, State University of New York, Buffalo, New York, United States of America; Julius-Maximilians-Universität Würzburg, Germany

## Abstract

**Background:**

Chronic cerebrospinal venous insufficiency (CCSVI) was described as a vascular condition characterized by anomalies of veins outside the skull was reported to be associated with multiple sclerosis (MS). The objective was to assess the associations between HLA DRB1*1501 status and the occurrence of CCSVI in MS patients.

**Methodology/Principal Findings:**

This study included 423 of 499 subjects enrolled in the Combined Transcranial and Extracranial Venous Doppler Evaluation (CTEVD) study. The HLA DRB1*1501 status was obtained in 268 MS patients and 155 controls by genotyping *rs3135005*, a SNP associated with DRB1*1501 status. All subjects underwent a clinical examination and Doppler scan of the head and neck. The frequency of CCSVI was higher (OR = 4.52, *p*<0.001) in the MS group 56.0% vs. 21.9% in the controls group and also higher in the progressive MS group 69.8% vs. 49.5% in the non-progressive MS group. The 51.9% frequency of HLA DRB1*1501 positivity (HLA^+^) in MS was higher compared (OR = 2.33, *p*<0.001) to 31.6% to controls. The HLA^+^ frequency in the non-progressive (51.6%) and progressive MS groups (52.3%) was similar. The frequency of HLA^+^ CCSVI^+^ was 40.7% in progressive MS, 27.5% in non-progressive MS and 8.4% in controls. The presence of CCSVI was independent of HLA DRB1*1501 status in MS patients.

**Conclusions/Significance:**

The lack of strong associations of CCSVI with HLA DRB1*1501 suggests that the role of the underlying associations of CCSVI in MS should be interpreted with caution. Further longitudinal studies should determine whether interactions between these factors can contribute to disease progression in MS.

## Introduction

Recently reported strong associations between MS and a condition defined as chronic cerebrospinal venous insufficiency (CCSVI), have challenged the prevailing view that central nervous system damage (CNS) in multiple sclerosis (MS) is predominantly the result of abnormal immune responses against the patient's nervous tissue [1], [2], [3].

CCSVI has been described as a vascular condition characterized by anomalies of the main extra-cranial cerebrospinal (CS) venous routes that interfere with normal CS venous outflow. These anomalies have been reported to affect the internal jugular veins (IJV), the vertebral veins (VV) and the azygous vein (AZY), and can be detected using venous echo-color Doppler (ECD) and catheter venography [1], [2], [3]. It has been hypothesized that CS venous anomalies may cause alterations to blood flow that eventually result in iron deposition, degeneration of neurons and characteristic brain injury patterns found in MS [4], [5]. Nevertheless, some studies have questioned the existence of the CCSVI in patients with MS [6], [7].

CCSVI is a controversial area in MS research and it is important to critically assess the role of CCSVI and its pathophysiological mechanisms so that the implications, if any, for the treatment and prevention of MS can be determined. However, the mechanisms that cause the reported associations between CCSVI and MS are not known. A valuable scientific step in this direction would be to place CCSVI in the context of other known associations in MS.

The genetics of MS has been systematically investigated in genomewide association studies (GWAS) [8], [9]. These studies have confirmed key associations with the MHC locus and identified additional genetic variations associated with the risk of developing MS [10]. Genetic epidemiology studies have also demonstrated that the genetics of MS is complex and involves interplay between genes and environmental factors. However, the genetic variations and the environmental factors do not individually explain the majority of the variance in the risk of developing MS [10], [11], [12]. There is suggestive evidence that genetic risk factors such as HLA DRB1*1501 and environmental risk factors such as Epstein-Barr virus (EBV) exposure and cigarette smoking are also associated with disease progression. Except for Ferlini et al. [13] who conducted preliminary analysis of copy number variations associated CCSVI in a group of 15 MS patients, no information is available on the role of genetic factors in CCSVI MS.

The goal of this study was to assess the associations of CCSVI with HLA DRB1*1501, a genetic variation that has been consistently linked to MS in familial and association studies.

## Methods

### Study Population

#### Study Design

This project utilized samples from the Combined Transcranial and Extracranial Venous ECD Evaluation (CTEVD study), which was designed to assess the prevalence of CCSVI in a large cohort of patients with MS, clinically isolated syndrome (CIS), healthy controls (HC) and controls with other neurological diseases (OND) using specific echo-color Doppler (ECD) criteria (see Supplementary Material in [14]). The CTEVD study enrolled a total of 499 subjects, including 289 MS, 21 CIS, 163 HC and 26 OND.

The participants received a clinical examination (not blinded) and an ECD scan of the head and neck (performed by a technician blinded to the subjects' diagnosis) [14]. Subjects also provided blood samples for genetic analysis that were also evaluated by a technician who was blinded to the subjects' disease or CCSVI status.

The study was approved by the University at Buffalo Human Subjects Institutional Review Board and all participants provided written informed consent.

### Echo-color Doppler Data Analysis

Cerebral venous return was examined by using the echo-color Doppler (ECD Esaote-Biosound My Lab 25) equipped with 2.5 and 7.5–10 Mhz transducers (Genoa, Italy), with the subject positioned on a tilt bed at 90° and 0° [2], [3].

The specific details of the length of exam, contraindications and limitations, subject assessment, examination guidelines, annotation documentation, specific Doppler parameters, criteria definitions, description of probes, positioning of the subject, techniques used, fulfilment of VH criteria and pathology definitions are provided elsewhere [14].

The presence of CCSVI was defined as the presence of two or more venous hemodynamic (VH) criteria as described in [14]. A subject was considered CCSVI-positive if ≥2 VH criteria were fulfilled. A subject was considered CCSVI-negative if <2 VH criteria were fulfilled. Subjects who were not assessed for some VH criterion, due to technical difficulty, were assumed not to have fulfilled that criterion. Subjects who fulfilled exactly one of the other 4 criteria and were not assessed on one VH criterion were classified CCSVI borderline; these individuals were conservatively categorized as CCSVI negative in the statistical analyses potentially biasing associations toward the null.

### Genotyping

HLA DRB1*1501 status was obtained by genotyping DNA from peripheral blood for *rs3135005*, a SNP strongly correlated with HLA DRB1*1501 status, using an allele discrimination kit (Assays-on-Demand genotyping kit, Applied Biosystems, Redwood City, CA). Genotyping was performed on a MX4000 (Stratagene) real-time thermal cycler and analyzed using the MX4000 software. Non-template controls produced negligible background signals.

We also amplified DNA fragments for 9 DNA samples (3 each of C/C, C/T and C/T genotypes) previously genotyped by allele discrimination (forward primer: 5′ TGC CTT TTA AAA TCC AAA GAC AT; reverse primer: 5′ AGA GCG AGA CCA GGA ACA AA) spanning the *rs3135005* C/T SNP [15]. PCR products were digested with *Afl* 11 restriction enzyme and then analyzed on an agarose gel. The agreement between the RFLP results and allele discrimination was 100% on the nine samples examined.

### Data Analysis

SPSS (SPSS Inc., Chicago, IL, version 15.0) statistical program was used for all statistical analyses.

Subjects with relapsing-remitting (RR) MS were categorized as non-progressive MS whereas those with relapsing and non-relapsing forms of secondary progressive (SP) and primary-progressive (PP) MS were categorized as progressive MS [16]. The homozygous *rs3135005* and heterozygous genotypes were categorized as DRB1*1501 positive whereas the homozygous wild type allele was categorized as DRB1*1501 negative.

One-way ANOVA followed by post-hoc independent sample *t*-tests were used to test for differences in means of continuous demographic variables such as age, age of onset, and disease duration. The chi-square test was used for analysis of count variables for categorical data and the Fisher exact test was used where appropriate.

Multinomial logistic regression with the Control-Non-progressive MS-Progressive MS status as the nominal dependent variable categories, age as a covariate and gender as a factor was also used to assess the role of CCSVI or HLA DRB1*1501. Analyses were conducted with main effects models containing either CCSVI or HLA DRB1*1501 and both CCSVI and HLA DR*1501. In addition, models containing an additional CCSVI *HLA DRB1*1501 interaction term were also assessed when significant main effects were observed for both CCSVI and HLA DRB1*1501.

To correct for multiple comparisons, a conservative Type I error level of 0.01 was used to assess significance; a trend was assumed if the Type I error level ≤0.10.

## Results

### Demographic and Clinical Characteristics

**Figure 1:**
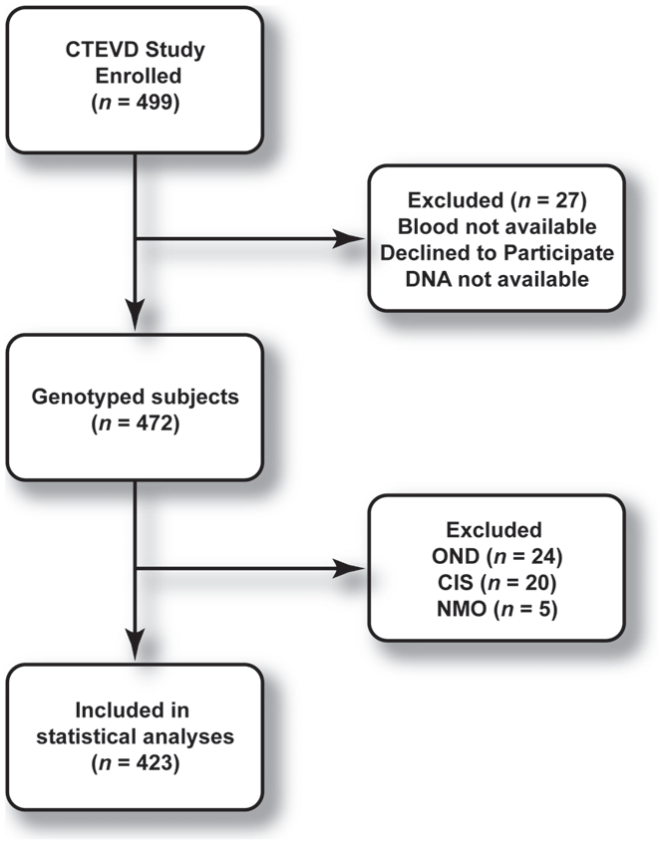
CONSORT flow chart for the study.

The CONSORT diagram for the study is summarized in Figure 1. Genotyping was available for 472 subjects. To avoid the effects of small samples and confusion stemming from three more groups, subjects with other neurological diseases (OND, *n* = 24), clinically isolated syndrome (CIS, *n* = 20) and neuromyelitis optica (NMO, *n* = 5) were excluded, yielding 423 subjects: 155 healthy controls and 268 CDMS in the statistical analysis. The comparisons were limited to healthy controls and patients with clinically definite MS according to the McDonald criteria [17].

Of the 268 MS patients, 182 had RRMS and 86 had progressive forms of MS. The clinical and demographic features of controls and MS patients are summarized in Table 1. There was a significant difference in the male to female ratio between MS cases and controls groups due to enrollment of spousal controls; analyses were adjusted for gender where appropriate.

**Table 1:** Demographic and clinical characteristics of the cohort.

Demographics	MS	Controls	*p*-value
Females: Males (% Female)	201: 67 (75%)	83*: 72 (54%)	<0.001‡
Disease course:Relapsing-remittingSecondary progressiveRelapsing SPPrimary progressive or primary relapsing	182 (67.9%)182 (67.9%)18 (6.7%)11 (4.1%)		
Race/Ethnicity:Caucasian-AmericanAfrican-AmericanHispanic/LatinoAsianOther/Unknown/Not given	243 (93.1%)13 (5.0%)4 (1.5%)0 (0%)1 (0.4%)	136 (89.5%)136 (89.5%)1 (0.7%)4 (2.6%)0 (0%)	0.20#
Age, years	46.4±12.1	44.8±14.1	0.24¶
Disease duration*, years	14.8±10.7	–	
Median EDSS* (IQR)	2.5 (4.0)		

The continuous variables are expressed as mean ± SD and the categorical variables as frequency (%).

*1 subject self-reported as transgender male, counted as a male.

# Fisher exact test for frequency of Whites to Non-whites in MS vs. Controls. For frequency of Black/African Americans to non-Black/African-Americans in MS vs. Controls *p* = 1.

‡ Fisher exact test.

¶ *t*-test.

### Frequency of CCSVI and HLA DRB1*1501 and CCSVI

The frequencies of CCSVI and HLA DRB1*1501 positive subjects are summarized in Table 2.

**Table 2:** The distribution of the HLA DRB1*1501 and CCSVI in MS and controls.

Demographics	DRB1*1501 Positive	CCSVI Positive
Controls	49/155 (31.6%)	34/155 (21.9%)
All MS	139/268 (51.9%)	150/268 (56.0%)
Non-progressive MS	94/182 (51.6%)	90/182 (49.5%)
Progressive MS	45/86 (52.3%)	60/86 (69.8%)

Odds ratios for DRB1*1501: For MS vs. Controls = 2.33 (95% CI: 1.54–3.53, *p*<0.001). For Non-progressive vs. Progressive MS = 1.03 (0.62–1.72, *p* = 1.0).

Odds ratios for CCSVI status: For MS vs. Controls = 4.52 (95% CI: 2.88–7.10, *p*<0.001). For Non-progressive vs. Progressive MS = 2.36 (1.37–4.07, *p* = 0.002).

The frequency for the homozygous HLA DRB1*1501 positive, heterozygous and homozygous HLA DRB1*1501 negative genotypes in MS patients were 18.7%, 33.2%, and 48.1%, respectively; the corresponding frequencies of these genotypes in controls were 5.8%, 25.8%, and 68.4%, respectively (chi-square = 20.6, *p*<0.001). The observed allele frequency for the disease-associated HLA DRB1*1501 allele in MS patients and controls were 35.3% and 18.7%, respectively. The odds ratio for the association between HLA DRB1*1501 positivity and the MS diagnosis was 2.33 (chi-square = 16.3, *p*<0.001). In multinomial logistic regression correcting for age and gender, there was no evidence for a significant association of HLA DRB1*1501 status (52.3% in progressive MS vs. 51.3% in non-progressive MS, *p* = 0.78) with non-progressive MS/progressive MS status.

The odds ratio for the association between CCSVI status and the MS diagnosis was 4.52 (chi-square = 46.2, *p*<0.001). In multinomial logistic regression correcting for age and gender, there was a significant association for CCSVI positivity (69.8% in progressive MS vs. 49.5% in non-progressive MS, *p* = 0.003) with non-progressive MS/progressive MS status.

We additionally conducted multinomial logistic regression correcting for age and gender with both CCSVI status and HLA DRB1*1501 status present among the predictor variables. CCSVI status was significantly associated with the Controls vs. Progressive MS (*p*<0.001) and Non-progressive vs. Progressive MS (*p* = 0.003) comparisons. There was trend toward an association between HLA DRB1*1501 status in the Controls vs. Progressive MS (*p* = 0.062) comparison but there was no evidence for a significant association in the Non-progressive vs. Progressive MS (*p* = 0.84) comparison.

The associations between HLA DRB1*1501 status and CCSVI status were significant when the entire study population was considered (chi-square = 10.3, Fisher exact test *p* = 0.002). However, there was no evidence for associations within the Control (chi-square = 0.88, Fisher exact test *p* = 0.41) sub-group and only a trend in the MS (chi-square = 3.15, Fisher exact test *p* = 0.085) sub-group. This suggests that the significant associations in the entire study population are largely the result of the indirect association or confounding of the HLA DRB1*1501 status and MS, which exhibits more CCSVI.

Table 3 summarizes the dependence of the Control/MS status and non-progressive MS/progressive MS status variables for different combinations of the HLA DRB1*1501 status and CCSVI status variables. The frequency of CCSVI negative- HLA DRB1*1501 negative status in Controls was more than two-fold greater than in MS patients (54.8% in Controls vs. 23.9% in MS), whereas the frequency of CCSVI positive- HLA DRB1*1501 positive status in MS patients (8.4% in Controls vs. 31.7% in MS) was more than three-fold greater than in Controls. The frequency of CCSVI positive- HLA DRB1*1501 positive status in the progressive MS sub-group was nearly four-fold greater than in Controls (8.4% in Controls vs. 40.7% in Progressive MS). Multinomial logistic regression with models containing both main effects and an interaction term between HLA DRB1*1501 status and CCSVI status variables did not provide evidence for a role for interactions.

**Table 3:** The joint distribution of the HLA DRB1*1501 status and CCSVI status.

	DRB1*1501 Negative		DRB1*1501 Positive	
Demographics	CCSVI Negative	CCSVI Positive	CCSVI Negative	CCSVI Positive
Controls	85 (54.8%)	21 (13.5%)	36 (23.2%)	13 (8.4%)
All MS	64 (23.9%)	65 (24.3%)	54 (20.1%)	85 (31.7%)
Non-progressive MS	48 (26.4%)	40 (22.0%)	44 (24.2%)	50 (27.5%)
Progressive MS	16 (18.6%)	25 (29.1%)	10 (11.6%)	35 (40.7%)

## Discussion

The goal of this study was to assess the associations of CCSVI with HLA DR*1501, a genetic variation that has been consistently linked to MS in familial and association studies. We found that the frequency of CCSVI positivity and HLA DRB1*1501 positivity were both increased in MS compared to controls. However, the frequency of CCSVI positivity was also increased in progressive forms of MS compared to the non-progressive forms of MS.

We reasoned that because HLA DRB1*1501 was well established as a genetic factor associated with the risk of developing MS, it would provide a reference relative to which the role of CCSVI could be evaluated. The goals were therefore to critically assess the associations of CCSVI with MS and MS progression vis-à-vis HLA DRB1*1501. We did not obtain evidence to support a role for statistical interactions between HLA DRB1*1501 and CCSVI status, which suggests that there is no synergistic association between HLA DRB1*1501 and CCSVI with MS. This is evidenced in non-progressive forms of MS because the relative proportions were the similar across the HLA DRB1*1501 negative-CCSVI negative, HLA DRB1*1501 positive -CCSVI negative, HLA DRB1*1501 negative-CCSVI positive, and HLA DRB1*1501 positive-CCSVI positive combinations. There was a higher relative frequency of the HLA DRB1*1501 positive-CCSVI positive combination compared to the HLA DRB1*1501 negative-CCSVI negative combination in progressive MS but this was not significant. The greater relative frequency of the HLA DRB1*1501 negative-CCSVI negative combination compared to the HLA DRB1*1501 positive-CCSVI positive combination in the control group could be interpreted as indicating that the absence of CCSVI is protective.

Although the association between susceptibility to MS and HLA-DRB1*1501 is well established, its relationship to disease characteristics and/or disease progression is controversial. Several studies have linked the DR2 haplotype to disease progression [18] especially if extreme cases (benign vs. malignant) are compared [19] but there is also evidence that a negative status for DRB1*1501 may be associated with a worse prognosis [20]. Our results however, did not provide support for a protective role for DRB1*1501 negative status in progressive MS status.

Interestingly, despite the lower prevalence of CCSVI in our sample compared to the results previously reported [2], the odds ratio for the association of CCSVI with MS was 4.52 compared to the odds ratio of 2.33 for the association of HLA DRB1*1501 with MS. Additionally, CCSVI positivity appeared associated with progressive forms of MS but we did not obtain evidence that HLA DRB1*1501 positivity was associated with progressive forms of MS in our sample. The exact reasons for the associations between CCSVI and progressive forms of MS are not known: only prospective longitudinal studies can address whether the associations are the result of CCSVI modifying disease progression or alternatively, because CCSVI is secondary to the underlying inflammatory/degenerative disease processes.

A potential criticism of our methodology is the use of ECD, which is sometimes viewed as technically demanding and strongly operator dependent. We used a single machine for all subjects and the one operator received extensive training in assessing CCSVI in MS; the operator's intra-rater reproducibility was Kappa 0.75 agreement with 89.3% in a scan-rescan test [14]. The operator was blinded to the subjects' clinical diagnosis and we included patients with OND because the obvious presence of disabilities in some patients adversely impacts the effectiveness of blinding [14]. Catheter venography and magnetic resonance venography are alternative imaging modalities capable of providing greater anatomical detail than ECD. However, these techniques are difficult to apply for the large sample sizes required for genetic analyses, e.g., the CV is an invasive exam and value of MRV for diagnosis of CCSVI is limited [21], [22], [23]. ECD provides qualitatively different functional assessments of flow velocity changes in response to postural adjustments that are complementary to, but not possible with the other imaging methods.

Other than the report of Ferlini et al. [13], who conducted preliminary analysis of copy number variations associated with CCSVI in a group of 15 MS patients, no information is available on the role of genetic factors in CCSVI. These authors reported that CCSVI was associated of copy number variations in the *HLA* region for a small group of 15 MS patients [13]. In other diseases with venous pathophysiologies, a role for gender, and environmental and genetic factors is suggested. Female gender, older age, and pregnancy are risk factors for chronic venous diseases [24] and women have greater frequency of variant hepatic veins [25]. Women have also been reported to have a smaller internal jugular vein size than men (1.48 for men vs. 1.27 in women) [26]. Venous malformations may have genetic contributions and a “double-hit” mechanism has been invoked to explain incomplete penetrance and variability [27], [28]. The R849W substitution in the angiopoietin receptor *Tie2* [29], an endothelial receptor tyrosine kinase, has been linked to familial venous malformations and results in variable thickness or lack of smooth-muscle cells in the veins of patient lesions. Interestingly Tie2 activates Stat1, which is also critical in interferon signaling. We did not observe, age, gender or disease duration differences in the occurrence of CCSVI (results not shown) in MS. However, a more detailed analysis of candidate gender-dimorphic factors, e.g., vein diameters and autoimmune factors, is warranted as these could strongly interact with changes in cerebral venous outflow.

HLA DRB1*1501 has been consistently linked to MS susceptibility in genetic studies. We did not find evidence for associations between CCSVI diagnosis and HLA DRB1*1501 status for MS patients.
